# Divergent Prognostic Value of Primary Tumor Segmentation Metrics on Baseline FDG PET/CT in Colorectal Cancer

**DOI:** 10.3390/cancers17213592

**Published:** 2025-11-06

**Authors:** Ken Kudura, Nando Ritz, Yves Schaulin, Arkadiusz Miszczyszyn, Tim Kutzker, Rebecca Engel, Marco von Strauss und Torney, Wolfgang Harms, Robert Foerster

**Affiliations:** 1 Department of Nuclear Medicine, University Hospital CHU UCL Godinne, 5530 Yvoir, Belgium; 2 Faculté de Médecine, Université Catholique de Louvain UCLouvain, 1348 Louvain, Belgium; 3 Faculty of Medicine, University of Basel, 4001 Basel, Switzerland; 4 Faculty of Medicine, Université Libre de Bruxelles (ULB), 1070 Brussels, Belgium; 5 Faculty of Applied Sciences, Humboldt University of Berlin, 10117 Berlin, Germany; 6 Department of Visceral Surgery, University Digestive Health Care Center, St. Clara Hospital and University Hospital Basel (Clarunis), 4058 Basel, Switzerland; 7 Department of Radiation Oncology, Sankt Clara Hospital, 4058 Basel, Switzerland; 8 Department of Radiation Oncology, Cantonal Hospital Winterthur, 8400 Winterthur, Switzerland; 9 Department of Radiation Oncology, Inselspital, Bern University Hospital, University of Bern, 3012 Bern, Switzerland

**Keywords:** 18F-FDG, PET/CT, colorectal cancer, tumor segmentation, outcome prediction

## Abstract

**Simple Summary:**

Colorectal cancer remains a biologically heterogeneous disease in which conventional staging fails to fully explain patient outcome variability. In this study, baseline FDG PET/CT was used to explore metabolic and morphological indicators capable of refining prognostic assessment. By quantifying metabolic tumor volume (MTV) and cranio-caudal extension, we identified two complementary imaging biomarkers that capture distinct aspects of tumor biology: MTV reflects systemic aggressiveness and metastatic potential, while cranio-caudal extension relates to local invasiveness and resectability. Integrating PET/CT-derived features into clinical evaluation could therefore enhance individualized treatment strategies, particularly in the era of multimodal therapies. This study reinforces the potential of metabolic imaging to move beyond anatomical staging and support a biologically driven approach to colorectal cancer management.

**Abstract:**

Background: Colorectal cancer (CRC) remains a major global health concern, with increasing incidence and mortality projected over the coming decades. Despite the central role of staging systems, substantial heterogeneity in clinical outcomes persists among patients within the same stage, highlighting the need for additional prognostic biomarkers. This study aimed to evaluate whether segmentation-derived morphological and metabolic features of the primary tumor could serve as prognostic biomarkers associated with subsequent tumor evolution in CRC. Methods: In this retrospective, single-center study, 91 patients with histologically confirmed CRC who underwent baseline FDG PET/CT prior to treatment were analyzed. Morphological (tumor shape, cranio-caudal extension, volume) and metabolic (SUVmean, SUVmax, MTV, TLG) parameters of the primary tumor were extracted using 3D segmentation. Clinical benefit (CB) was defined according to RECIST criteria at six months. Logistic regression and Cox proportional hazards models were applied to identify predictors of short- and long-term outcomes, with performance assessed using ROC curves and Kaplan–Meier survival analyses. Results: Cranio-caudal extension was the strongest prognostic biomarker of short-term clinical benefit (AUC = 0.89), with a threshold of 6.2 cm discriminating favorable from unfavorable outcomes. In multivariate analysis, early UICC stage and lower cranio-caudal extension were independently associated with CB. For long-term outcomes, MTV emerged as a consistent prognostic factor: higher MTV predicted shorter progression-free survival (HR = 1.03, *p* < 0.01) and overall survival (HR = 1.03, *p* < 0.01). In addition, UICC stage IV significantly increased the risk of progression (HR = 9.65, *p* < 0.01). Conclusions: Segmentation of the primary tumor on baseline FDG PET/CT provides valuable prognostic information in CRC. While cranio-caudal extension was the strongest prognostic biomarker of short-term treatment response, MTV was independently associated with long-term outcomes, particularly progression-free survival. These findings highlight the complementary prognostic roles of morphological and metabolic tumor features and support the integration of PET/CT-based biomarkers into personalized treatment strategies for colorectal cancer.

## 1. Introduction

Colorectal cancer (CRC) continues to represent a significant global health challenge. In 2020, more than 1.9 million new cases were diagnosed worldwide, leading to over 930,000 deaths and establishing CRC as the second most common cause of cancer-related mortality [1]. Alarmingly, projections for 2040 anticipate a 63% increase in annual incidence, culminating in approximately 3.2 million new cases, and a 73% increase in mortality, with an estimated 1.6 million deaths per year [1]. At initial diagnosis, between 15% and 30% of patients already present with metastatic disease, and approximately 20% of those treated for localized tumors subsequently develop distant metastases [2]. Survival outcomes vary substantially according to disease stage, ranging from approximately 90% in localized CRC to less than 10% in cases of metastatic dissemination [3]. However, significant heterogeneity in clinical outcomes persists even among patients classified within the same stage, underscoring the urgent need for additional prognostic markers to refine risk stratification and optimize therapeutic strategies [4]. In recent years, therapeutic paradigms in CRC have evolved considerably, notably with the introduction of neoadjuvant treatment protocols, which have contributed to improved oncological outcomes [5,6,7]. Nonetheless, these advances concurrently raise critical questions regarding the optimal identification of patients most likely to benefit from specific therapeutic interventions.

Fluorodeoxyglucose positron emission tomography combined with computed tomography (18F-FDG PET/CT) has become a cornerstone modality in oncologic imaging. According to the 2023 guidelines of the European Society for Medical Oncology (ESMO), FDG PET/CT is recommended for staging when elevated tumor markers are present without an identifiable metastatic site and may assist in evaluating the resectability of suspected metastases [8]. Despite its superior sensitivity compared to conventional computed tomography (CT) for detecting lymph node and distant metastases, FDG PET/CT is not currently endorsed for routine initial staging of CRC [8]. FDG PET/CT enables comprehensive quantitative assessment of tumor metabolism through parameters such as standardized uptake value (SUV), metabolic tumor volume (MTV), and total lesion glycolysis (TLG). A growing body of evidence suggests that both MTV and TLG are independent prognostic biomarkers in CRC, providing critical information complementary to traditional clinical and pathological factors [9,10,11,12,13]. In a recent investigation conducted by our group, we demonstrated that FDG PET/CT exhibits superior diagnostic accuracy compared with conventional imaging modalities for initial staging of colorectal cancer, leading to substantial alterations in therapeutic management strategies in a considerable proportion of cases [14]. These findings highlight the potential for FDG PET/CT to play an increasingly central role in the initial assessment of CRC.

Accordingly, the present study aimed to evaluate whether morpho-metabolic features derived from baseline FDG PET/CT segmentation of the primary tumor could serve as prognostic biomarkers associated with subsequent tumor evolution in colorectal cancer patients treated with standard therapy. Rather than assessing treatment efficacy, this study sought to identify imaging features potentially reflective of the tumor’s intrinsic biological behavior.

## 2. Methods

### 2.1. Study Design and Patient Population

This retrospective, single-center study received approval from the Ethics Committee for Northwest and Central Switzerland (EKNZ-Nr: 2022-00248) and was carried out in compliance with Good Clinical Practice (GCP) guidelines and the Declaration of Helsinki. All patients included in this study had signed the institutional general consent form routinely provided, authorizing the use of their anonymized clinical and imaging data for research purposes.

We retrospectively screened our Tumor Reference Center database for adult patients diagnosed with colorectal cancer between 2020 and 2021. Eligibility required the availability of a baseline FDG PET/CT performed prior to any treatment. Patients with incomplete clinical or imaging data were excluded from the analysis.

### 2.2. Data Collection

All clinical (Section 2.2.1), imaging (Section 2.2.2), and follow-up data were systematically collected from sources including medical records, tumor board recommendations, imaging protocols, and laboratory data, and subsequently compiled into a standardized database.

#### 2.2.1. Clinical Data

For all included patients, we collected clinical data including age, gender, localization of the primary tumor, stage according to union for international cancer control (8th version of UICC), microsatellite instability-, BRAF- and KRAS-status, progression-free survival (PFS), overall survival (OS), and date of death. OS has been defined as the time from the date of diagnosis to death from any cause or last follow-up, while PFS as time from the date of diagnosis to documented disease progression or death, whichever occurred first.

#### 2.2.2. Imaging Data Analysis

For all included patients, we collected relevant imaging parameters from baseline FDG PET/CT performed prior to any treatment. Patient-related acquisition parameters, including blood glucose level and body mass index (BMI), were recorded to ensure appropriate scan quality. Imaging analyses focused on both the primary tumor and the presence and location of metastases, with metastatic sites categorized as lung, liver, thyroid, colon, kidney, soft tissue, bone, or other. The primary tumor location was classified according to colonic segment: ascending, transverse, or descending colon.

According to the Nuclear Medicine department protocol, all PET/CT scans were routinely reviewed by two physicians, each specialized in both radiology and nuclear medicine.

#### 2.2.3. Primary Tumor Segmentation

Quantitative analysis of the primary colorectal tumor on baseline FDG PET/CT prior to any treatment was performed using a 3D contouring tool (advanced workstation version 4.7, GE, Milwaukee, WI, USA). Scans were reviewed in all planes, with CT images used independently or fused with PET for accurate localization. Lesions were carefully delineated to exclude physiological or unrelated uptake, with a minimum ROI volume of 1 mL. Manual refinement was applied for small or low-contrast primary tumors. All segmentations were independently reviewed by two physicians, and discrepancies were resolved by consensus. Contrast medium was not systematically used.

For the primary tumor, both morphological and metabolic parameters were extracted. Morphological features included tumor shape (circular or semi-circular), cranio-caudal extension (in cm), and overall tumor volume (in cm^3^). Metabolic parameters comprised mean/maximum standardized uptake value (SUVmean/SUVmax), metabolic tumor volume (MTV in cm^3^ with a 41% SUVmax threshold), and total lesion glycolysis (TLG).

#### 2.2.4. Short-Term Response Assessment

Treatment response was assessed on imaging performed on average six months after treatment initiation and patients were categorized into two distinct groups: clinical benefit (CB), defined as complete response, partial response, or stable disease, and no clinical benefit (no-CB), defined as progressive disease, according to the response evaluation criteria in solid tumors (RECIST) criteria.

#### 2.2.5. Outcomes

The primary outcome was the identification of prognostic factors among the aforementioned parameters in relation to clinical benefit in the short term, but also to overall survival (OS) and progression-free survival (PFS) in the long term.

The secondary outcome was to establish threshold values for parameters deemed significant, enabling the classification of patients according to their risk of unfavorable disease progression.

### 2.3. Statistical Analysis

Patients and segmented primary tumors characteristics were summarized using descriptive statistics, with absolute numbers and percentages for categorical variables and medians for continuous variables. Categorical variables were compared using the Chi-squared test, and continuous variables using Student’s *t*-test.

Predictive factors of CB were identified by first performing variable selection through forward selection, followed by inclusion in a multivariable logistic regression to determine factors independently associated with CB. For continuous variables, optimal cut-off values were determined iteratively as those best discriminating between CB and no-CB groups, testing values between the 10th and 90th percentiles. The performance of these cut-offs was assessed using receiver operating characteristic (ROC) curve analysis.

The impact of predictive factors on PFS and OS was evaluated using Cox proportional hazards regression, followed by multivariable Cox regression to identify independent prognostic factors. Kaplan–Meier survival curves were generated to visualize the observed outcomes. Statistical significance was defined as *p* < 0.05 for all analyses.

## 3. Results

### 3.1. Patient Population

**Figure 1:**
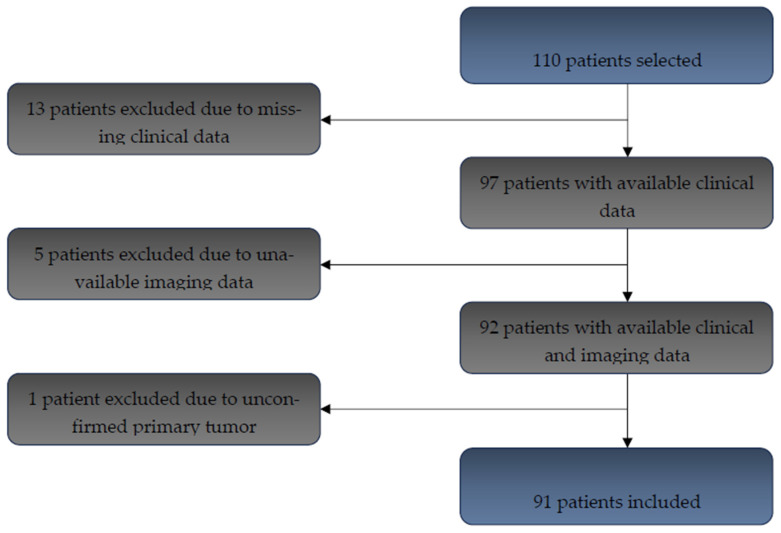
Flow chart for patient inclusion (*n* = 91 included patients).

A total of 110 adult patients diagnosed with histologically confirmed colorectal cancer between 2020 and 2021 were initially identified in our database. Thirteen patients were excluded due to missing clinical data, five due to unavailable imaging, and one due to lack of histological confirmation of the primary tumor. After applying these exclusion criteria, 91 patients were included in the final analysis (Figure 1).

The study population included 91 patients, of whom 70 experienced clinical benefit (CB) and 21 did not (no-CB). The median age was 71 years (range 62–79) in the CB group and 72 years (range 52–76) in the no-CB group, with a similar gender distribution (69% male in CB vs. 62% in no-CB). Primary tumors were most frequently located in the rectum (67.1% CB vs. 52.4% no-CB), followed by the sigmoid colon (17.1% vs. 33.3%), with no significant difference between groups (*p* = 0.26). Regarding metastases, 77.1% of CB patients and 71.4% of no-CB patients were non-metastatic at baseline (*p* = 0.14). UICC stage distribution differed significantly, with the majority of CB patients at stages I–III (87.1%) and most no-CB patients at stage IV (71.4%; *p* < 0.01) Table 1.

### 3.2. Primary Tumor Metabolic and Morphological Parameters

Metabolic parameters measured on baseline FDG PET/CT of the segmented primary tumor are summarized in Table 2. MTV differed significantly between groups, with a median of 10.7 cm^3^ (6.4–19.3) in CB patients compared to 20.9 cm^3^ (15.7–36.4) in no-CB patients (*p* = 0.01), whereas SUVmax, SUVmean and TLG showed no significant differences between the groups.

Morphological parameters of the segmented primary tumor are summarized in Table 3. CB patients had significantly smaller tumors, with shorter craniocaudal extension (median 4.4 vs. 6.1 cm, *p* = 0.01) and lower volume (median 10.8 vs. 21.1 cm^3^, *p* = 0.01), while tumor shape also differed between groups (*p* < 0.01).

### 3.3. Prognostic Biomarkers for Treatment Response

#### 3.3.1. Treatment Characteristics

**Figure 2:**
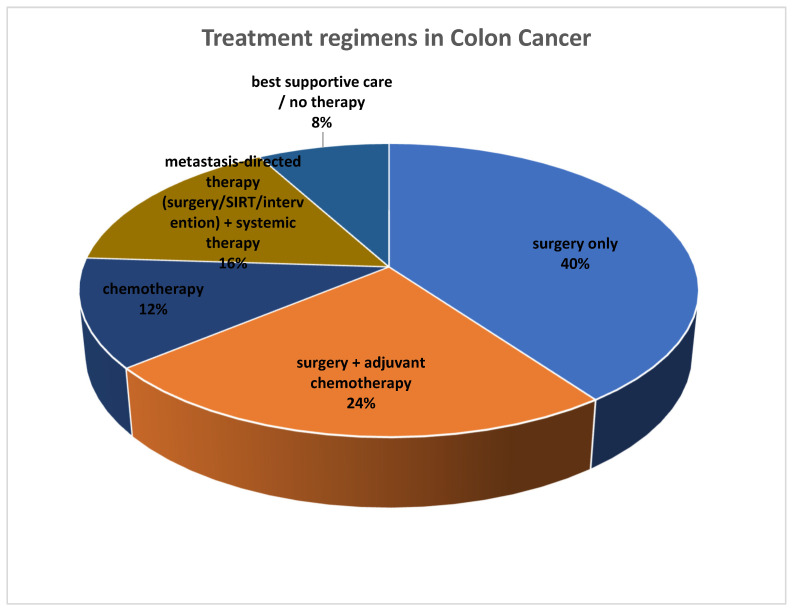
Overview of treatment regimens in patients with colon cancer (*n* = 25).

**Figure 3:**
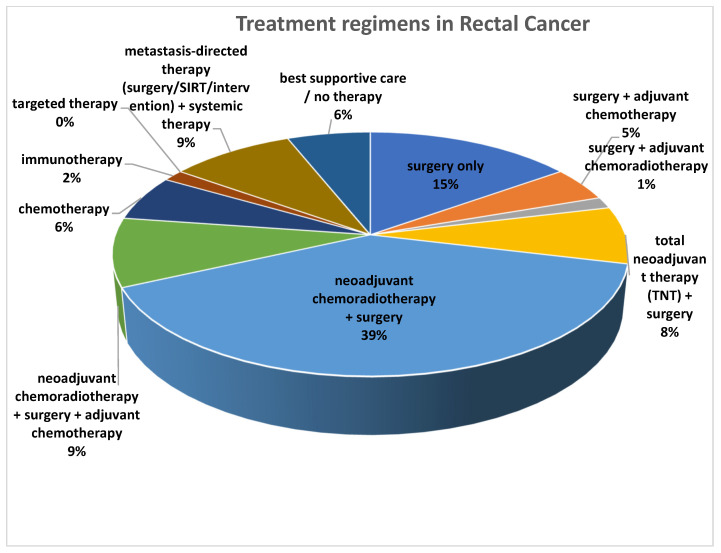
Overview of treatment regimens in patients with rectal cancer (*n* = 66).

Before assessing the prognostic value of the imaging parameters, we first detailed the treatment regimens. Treatments are reported separately for patients with colon cancer (Figure 2) and those with rectal cancer (Figure 3).

Among the 25 patients with colon cancer included in the study, the majority underwent surgery, either alone (*n* = 10) or combined with chemotherapy (*n* = 6). A smaller group received chemotherapy alone (*n* = 3) or metastasis-directed therapy with systemic treatment (*n* = 4). Two patients received only supportive care or no therapy (*n* = 2), as displayed in Figure 2.

Among the 66 rectal cancer therapies recorded, the most frequent approach was neoadjuvant chemoradiotherapy followed by surgery (*n* = 26). Surgery alone (*n* = 10) or combined with chemotherapy (*n* = 3) was also common, while immunotherapy and targeted therapy were rarely used. A small number of patients received only supportive care or no therapy, as displayed in Figure 3.

#### 3.3.2. Short-Term Response

In the first step of the statistical analysis, we performed univariable logistic regression to identify which parameters of the primary tumor were predictive of CB. All the aforementioned clinical, morphological, and metabolic parameters were considered for the univariable logistic regression analysis. For each parameter, the model provided the odds ratio (OR), log-likelihood (LL), null log-likelihood (LL-null), and pseudo-R^2^ as measures of goodness of fit.

Among all evaluated features, the morphological parameters including primary tumor shape (circular vs. semi-circular), craniocaudal extension, and tumor volume were showing a significant association with clinical benefit. The OR indicates the magnitude of association between each parameter and the probability of CB response, while the pseudo-R^2^ and the difference between LL and LL-null provide an estimate of the explanatory power of the model.

Craniocaudal extension emerged as the strongest predictor. Patients with shorter craniocaudal extension demonstrated a significantly higher likelihood of CB (median 4.4 cm vs. 6.1 cm, *p* = 0.01). This parameter yielded the highest pseudo-R^2^ (0.29) and the largest improvement in model fit (log-likelihood −28.1 vs. LL-null −39.6), underscoring its superior discriminative ability. The OR (0.42, 95% CI 0.22–0.80) further indicated that each unit increase in craniocaudal length was associated with a substantially decreased probability of CB.

In comparison, tumor morphology (circular vs. semi-circular) was also significantly associated with CB (*p* < 0.01, OR = 6.5), but the explanatory power of the model remained lower (pseudo-R^2^ = 0.17, LL −32.4 vs. LL-null −39.6). Similarly, primary tumor volume was predictive (median 10.8 cm^3^ vs. 21.1 cm^3^, *p* = 0.01, OR = 0.68), but again showed weaker fit indices (pseudo-R^2^ = 0.14, LL −33.1 vs. LL-null −39.6).

Altogether, these findings demonstrate that although several morphological parameters were significantly associated with CB, craniocaudal extension provided the highest predictive performance across all statistical indices, therefore representing the most robust morphological biomarker in this setting.

A forward selection procedure was performed to select variables for inclusion in a multivariate model, resulting in the identification of four parameters: age, sex, UICC stage, and craniocaudal extension. A multivariate logistic regression analysis was then conducted to identify factors independently associated with clinical benefit. Hazard ratios were subsequently calculated from the regression coefficients. This analysis showed that early UICC stage (HR = 0.06, 95% CI: 0.02–0.23) and lower craniocaudal extension (HR = 0.74, 95% CI: 0.57–0.96) were significantly associated with a better therapeutic response. The other variables tested did not reach statistical significance (Table 4).

The optimal cut-off for the cranio-caudal extension was determined through an iterative approach, testing values between the 10th and 90th percentiles, and selecting the threshold that maximally discriminated between the two groups in terms of clinical benefit. It was determined that patients with a craniocaudal extension of the primary tumor greater than 6.2 cm were more likely to have an unfavorable outcome.

**Figure 4:**
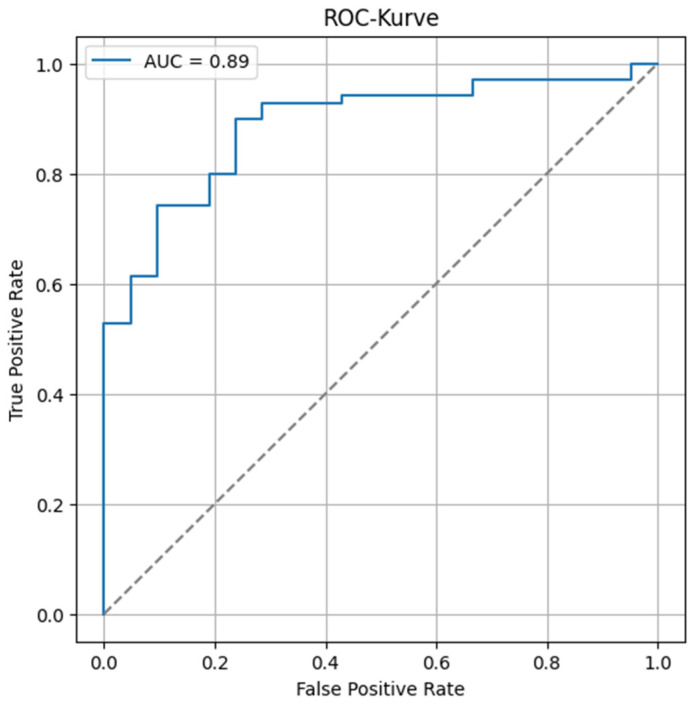
Receiver operating characteristic curve of the model’s performance to predict clinical benefit. The dashed line corresponds to an area under the curve of 0.5, representing a random phenomenon.

The overall predictive performance of the model was assessed using a Receiver Operating Characteristic (ROC) curve. The area under the curve (AUC) was calculated to evaluate the model’s ability to discriminate between positive and negative outcomes. The resulting AUC of 0.89 indicates excellent discriminative performance (Figure 4).

**Figure 5:**
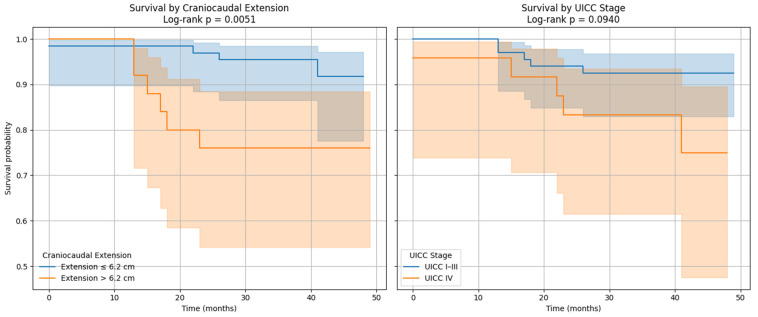
Kaplan–Meier survival curves for craniocaudal extension and UICC stage cut offs.

Survival curves were generated and confirmed that patients with UICC stage I–III and those with a cranio-caudal extension of the primary tumor less than 6.2 cm had better survival (Figure 5).

#### 3.3.3. Long-Term Response

We first investigated the association between the previously described clinical and imaging parameters and long-term outcomes, including both overall survival (OS) and progression-free survival (PFS), by performing univariate Cox regression analyses. Variables with at least one *p*-value below 0.2 were subsequently considered for multivariable testing, while highly correlated variables (r > 0.6) were excluded to avoid collinearity.

Patients with CB had significantly longer OS and PFS compared to those without CB (OS: 36.1 vs. 29.1 months, *p* = 0.03; PFS: 36.1 vs. 14.4 months, *p* < 0.01). Observation time was similar between groups (36.6 vs. 37.6 months, *p* = 0.58), while the mortality rate was significantly lower in the CB group (0.0% vs. 48.0%, *p* < 0.01), as captured in Table 5.

##### Univariate Cox Regression Analysis for Overall Survival (OS)

In the univariate models in Table 6, UICC stage IV, craniocaudal tumor extension, and primary tumor MTV each show a trend toward poorer OS, though without reaching statistical significance (HR = 2.76, *p* = 0.11; HR = 1.20, *p* = 0.10; and HR = 1.02, *p* = 0.05, respectively). Male patients tended to have longer survival than females (HR = 0.29, *p* = 0.06). The lack of statistical significance is likely explained by the limited number of events observed for OS (number of deaths 10).

##### Univariate Cox Regression Analysis for Progression-Free Survival (PFS)

In contrast, the PFS analyses displayed in Table 7 reveal stronger associations. UICC stage IV was identified as the strongest predictor, with patients in this group showing nearly a nine-fold higher risk of disease progression compared with those in stages I–III (HR = 9.37, *p* < 0.01). Craniocaudal extension was also a significant predictor (HR = 1.26, *p* < 0.01), as was MTV of the primary tumor (HR = 1.02, *p* < 0.01). Sex showed no association with PFS (HR = 0.71, *p* = 0.45).

##### Multivariate Cox Regression Analysis for OS and PFS

Multivariable Cox models were then constructed using forward regression, while applying the rule of at least 10 events per predictor. A total of 16 models were initially tested, and two were selected based on Akaike Information Criterion (AIC) and concordance index values, as captured in Table 8.

For OS, the model including sex and MTV of the primary tumor provided the best performance (AIC = 81.81; concordance index = 0.77). In this analysis, female sex was independently associated with worse OS (HR = 0.17, *p* = 0.02), while higher MTV remained a significant negative prognostic factor (HR = 1.03, *p* < 0.01).

For PFS, the optimal model retained UICC stage and MTV (AIC = 153.83; concordance index = 0.82). Here, stage IV disease conferred an almost ten-fold increased risk of progression (HR = 9.65, *p* < 0.01), and higher MTV was again independently associated with poorer outcome (HR = 1.03, *p* < 0.01).

##### Kaplan–Meier Survival Curves

**Figure 6:**
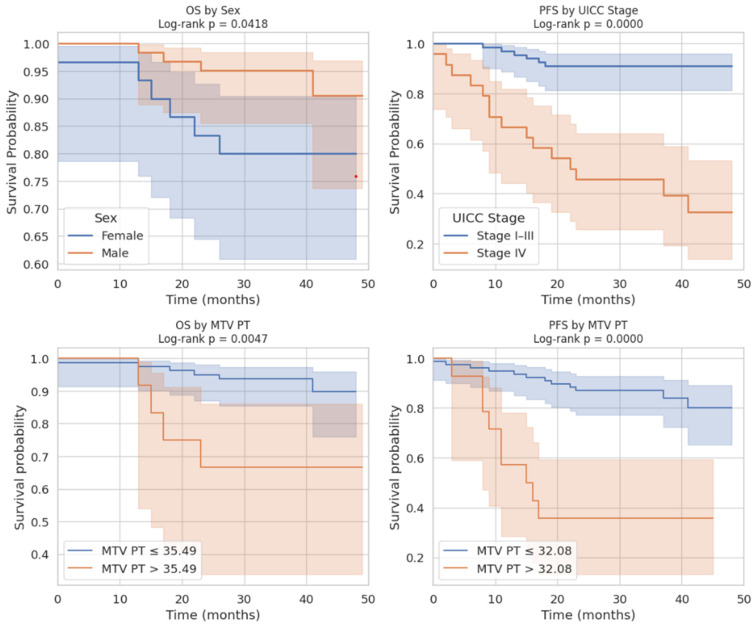
Integrated visualization of Kaplan–Meier survival analyses for features predicting long-term outcome.

The Kaplan–Meier survival analyses illustrated these associations. For OS, patients with MTV values below the optimal cut-off of 35.49 had significantly better survival outcomes. In addition, male patients demonstrated a tendency toward longer survival compared to females. For PFS, patients above the MTV cut-off of 32.08 had a significantly shorter time to progression as captured in Figure 6. Finally, stratification by UICC stage showed that patients with stage IV colorectal cancer had the poorest PFS, confirming the results of the Cox regression.

**Figure 7:**
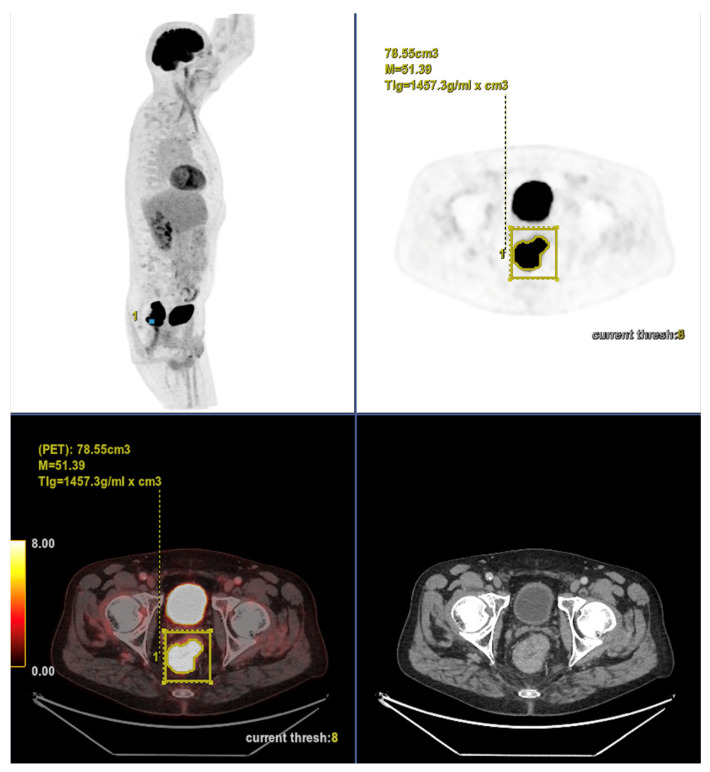
Representative example of manual delineation of a primary rectal tumor on baseline FDG PET/CT showing manual delineation of the metabolically active tumor volume (**first row**) on the PET image with corresponding fused PET/CT image and CT image with iodinated contrast medium (**second row**). Morphological features, including tumor shape (circular or semi-circular), cranio-caudal extension (in cm), and overall tumor volume (in cm^3^), were assessed on the CT image (often with contrast medium) coregistered with the PET images. Tumor shape was determined on the axial plane, cranio-caudal extension on the sagittal plane, and tumor volume after careful delineation on all planes. Iodinated contrast medium was administrated in 70% of cases after exclusion of contraindications. Metabolic parameters, including SUVmax, SUVmean, MTV and TLG, were systematically extracted from the segmented tumor (yellow in this figure). SUVmax (M in this figure) and TLG were automatically displayed. SUVmean could be obtained by simply replacing SUVmax with SUVmean in the parameter selection. MTV was automatically calculated from TLG and SUVmean values displayed on the workstation. The highlighted region in yellow represents the 3D contour of the primary tumor, excluding physiological uptake and non-tumor regions.

Altogether, these analyses demonstrate that MTV of the primary tumor is a consistent predictor of both OS and PFS. While OS was primarily influenced by sex and MTV, PFS was more strongly determined by UICC stage and MTV, as displayed in Figure 6. For a clearer understanding of the presented results, Figure 7 shows a representative example of manual delineation of the primary tumor on baseline FDG PET/CT.

Case 1: An 80-year-old patient with stage IVA rectal cancer and synchronous anal carcinoma, presenting with a large circumferential primary tumor showing a craniocaudal extension well above the predictive cut-off (13.0 cm vs. 6.2 cm; volume: 79.2 mL) and a metabolic tumor volume markedly exceeding both prognostic thresholds (MTV 79.0 vs. 35.49 for OS and 32.08 for PFS). Baseline FDG PET/CT demonstrates locoregional nodal involvement and a solitary liver metastasis in segment VII. The patient experienced rapid disease progression, with a progression-free survival of 8 months and an overall survival of 27 months.


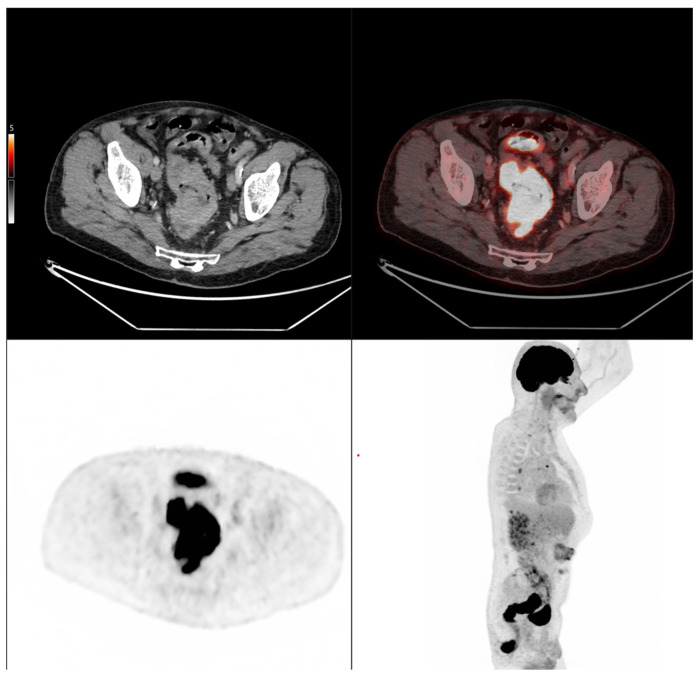


Case 2: A 69-year-old patient with stage IIA rectal cancer, presenting with a semi-circumferential primary tumor with craniocaudal extension and MTV both below the predictive cut-offs (5.1 cm vs. 6.2 cm; MTV 10.1 vs. 35.49 for OS and 32.08 for PFS). Baseline FDG PET/CT (top row) revealed intense hypermetabolism (SUVmax 38.8). Interim FDG PET/CT (bottom row) showed a good partial metabolic response, and the patient experienced sustained clinical benefit with no progression in the long term (OS = PFS = 29 months).


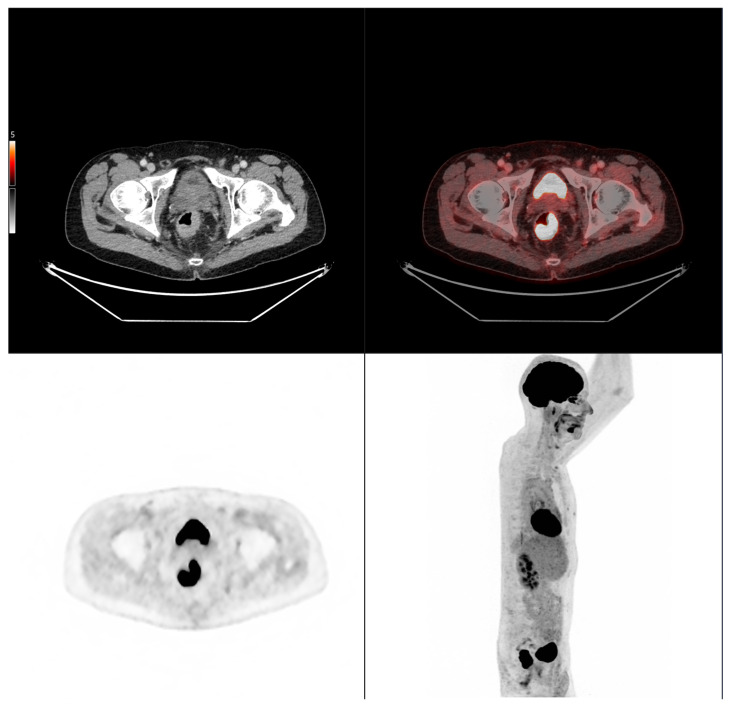



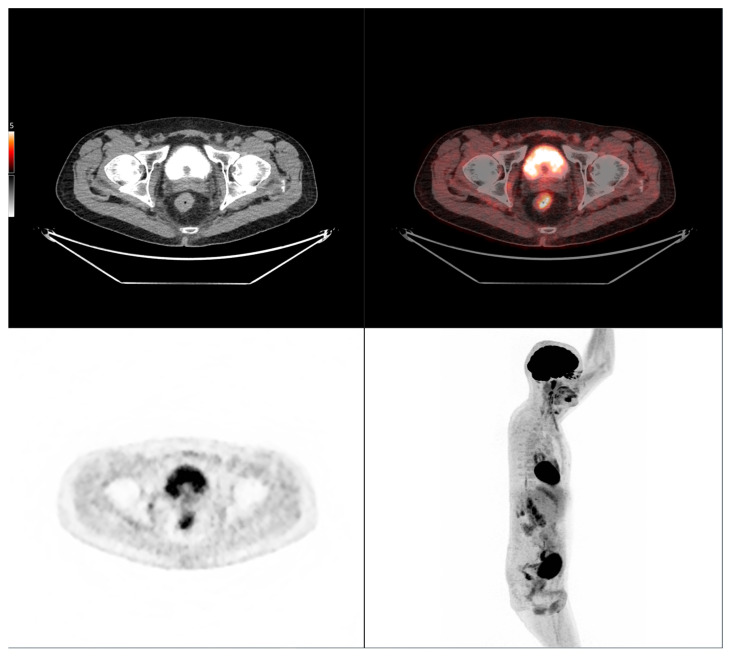


## 4. Discussion

Colorectal cancer (CRC) remains one of the most pressing global oncologic challenges, with a steadily increasing incidence and mortality burden. While current staging systems are fundamental for clinical management, they fall short in accounting for the substantial heterogeneity in outcomes among patients within the same stage [4]. Accordingly, the present study focused on evaluating whether baseline FDG PET/CT features of the primary tumor could serve as prognostic biomarkers in colorectal cancer patients treated with standard therapy. The aim was to explore imaging characteristics potentially reflective of intrinsic tumor behavior, rather than to assess treatment efficacy.

We demonstrated that cranio-caudal extension was the strongest prognostic biomarker of short-term treatment response, as defined by clinical benefit (CB), with a high discriminative performance (AUC of 0.89). This finding highlights the potential of morphological tumor burden, easily measured from standard PET/CT acquisitions, to guide early clinical decisions. In contrast, MTV was not associated with CB, but showed a significant correlation with progression-free survival (PFS), confirming its role as a prognostic biomarker of systemic tumor aggressiveness. This dual prognostic behavior is biologically coherent. Cranio-caudal extension likely reflects local invasiveness and resectability, two factors closely tied to immediate treatment outcomes [13]. Conversely, MTV captures the global glycolytic activity of the tumor, which may indicate biological aggressiveness and a higher likelihood of dissemination. The stronger association of MTV with PFS than with overall survival (OS) aligns with prior studies reporting that metabolic burden predicts early relapse but may not fully translate into reduced OS due to salvage therapies and treatment heterogeneity [9,10,11,12,13,15,16,17].

Importantly, our findings take on added significance in the context of the shifting therapeutic paradigm in CRC. The adoption of neoadjuvant treatment protocols, especially for rectal cancer and increasingly for selected colon cancers, reflects a move toward earlier systemic intervention and organ preservation [14,15,16,17,18,19]. In this setting, early imaging biomarkers that predict subsequent tumor evolution under standard of care prior to therapy are critical. Our findings indicate that cranio-caudal extension and MTV, derived from a single baseline FDG PET/CT scan, are associated with tumor evolution and may help stratify patients according to the intrinsic aggressiveness of their disease. These imaging parameters could inform clinical decision-making regarding treatment intensity, timing of surgery, and follow-up strategies. Such prognostic insights are particularly relevant in the context of precision oncology, where integrating imaging and molecular biomarkers can enhance patient-tailored management.

Recent studies have further expanded the prognostic relevance of PET/CT in CRC. Beyond traditional metabolic indices, PET/CT-derived features have been linked to microsatellite instability status, which holds significant implications for prognosis and immunotherapy eligibility [20]. Additionally, radiomic and texture analyses extracted from FDG PET/CT enhance risk stratification, particularly in stage II disease, and in some studies outperform conventional T-stage in predicting survival [21,22,23]. Quantitative indices such as TLG and measures of intratumor metabolic heterogeneity have also been shown to provide incremental prognostic information for recurrence and disease-free survival [24,25,26]. Recently published investigations have demonstrated that early metabolic changes on PET/CT can predict pathologic response and long-term outcomes following neoadjuvant therapy [18,19,20,21,22,23,24,25]. Moreover, inflammatory markers derived from FDG uptake in the bone marrow and spleen have recently been identified as additional prognostic indicators linked to systemic immune and inflammatory responses [27].

An important aspect of our findings lies in the distinct prognostic roles observed for morphological and metabolic features on pre-treatment FDG PET/CT. While various previous studies have focused either on metabolic parameters such as SUV, MTV, or TLG, or on morphological descriptors in isolation, our analysis demonstrates that these two dimensions do not behave uniformly [9,10,11,18,19,28,29]. This temporal dissociation is, to our knowledge, novel and highlights the complementary value of morphology and metabolism in understanding tumor behavior. Furthermore, a notable methodological aspect of our study is the decision to segment only the primary tumor. This choice was both pragmatic and justified: fewer than 25% of patients in our cohort had metastatic disease, suggesting that the primary lesion accounts for the bulk of tumor burden in most cases. In fact, primary tumor segmentation is significantly faster, more robust, and clinically implementable than whole-body segmentation, which requires advanced software and trained personnel. While excluding nodal and distant metastases may underestimate the total tumor burden in advanced disease, this limitation is likely to have had a modest impact in our predominantly non-metastatic cohort. Nevertheless, future studies should assess whether incorporating whole-body volumetric data enhances predictive accuracy in more advanced stages.

From a methodological standpoint, our iterative approach to cut-off determination, testing values between the 10th and 90th percentiles, adds robustness by avoiding arbitrary threshold selection and maximizing discrimination between groups.

Finally, we focused on stratification prior to any treatment initiation. This design distinguishes our work from several earlier studies that primarily examined patients who were either refractory to chemotherapy or analyzed outcomes in the post-surgical setting [10,11,29,30]. By evaluating patients at baseline, we aimed to capture the intrinsic prognostic value of morphological and metabolic tumor features before any potential modification induced by systemic or local therapies.

However, several limitations must be acknowledged. First, the retrospective single-center design may limit generalizability. Second, variability in acquisition protocols (including the use of contrast medium), and thresholding methods (41% SUVmax threshold used) may introduce measurement bias. Third, our use of clinical benefit as a binary endpoint, while clinically meaningful, may oversimplify heterogeneous biological responses. Additionally, the cohort included patients who received different treatment regimens, including neoadjuvant, adjuvant, and palliative strategies. Given the limited sample size, subgroup analyses based on treatment modality were not feasible. These issues underscore the need for prospective multicenter studies with standardized imaging and therapeutic protocols to validate the clinical significance and practical application value of the current findings.

Finally, our results contribute to the growing body of research supporting the integration of FDG PET/CT into initial staging and risk stratification in CRC, even when distant metastases are not present. Current ESMO guidelines restrict routine PET/CT use to specific clinical scenarios [5,6,7,8], but our findings support its broader application as a source of actionable prognostic information, especially when combined with clinical markers.

## 5. Conclusions

In conclusion, baseline FDG PET/CT was used to explore metabolic and morphological indicators capable of refining prognostic assessment. By quantifying metabolic tumor volume and cranio-caudal extension, we identified two complementary imaging biomarkers that capture distinct aspects of tumor biology: MTV reflects systemic aggressiveness and metastatic potential, while cranio-caudal extension relates to local invasiveness and resectability. Integrating PET/CT-derived features into clinical evaluation could therefore enhance individualized treatment strategies, particularly in the era of multimodal therapies. This study reinforces the potential of metabolic imaging to move beyond anatomical staging and support a biologically driven approach to colorectal cancer management.

## Figures and Tables

**Table 1:** Baseline patient characteristics (*n* = 91) stratified by clinical benefit (CB, *n* = 70) vs. no clinical benefit (No-CB, *n* = 21).

	CB (*n* = 70)	No-CB (*n* = 21)	*p*-Value
Age, years median (range)	71 (62–79)	72 (52–76)	0.18
Gender, *n* male/female (%)	48/22 (69/31)	13/8 (62/38)	
Localization of the primary tumor, *n* (%)			0.26
- Rectum	47 (67.1)	11 (52.4)	
- Sigmoid colon	12 (17.1)	7 (33.3)	
- Ascending colon	5 (7.1)	3 (14.3)	
- Transverse colon	4 (5.7)	0 (0)	
- Descending colon	2 (2.9)	0 (0)	
Anatomical site of metastases, *n* (%)			0.14
- Non metastatic	54 (77.1)	15 (71.4)	
- Kidney	3 (4.3)	1 (4.8)	
- Colon	1 (1.4)	1 (4.8)	
- Thyroid	1 (1.4)	0 (0)	
- Breast	0 (0)	2 (9.5)	
- Other	11 (15.7)	2 (9.5)	
UICC stage, *n* (%)			<0.01
- I	13 (18.6)	0 (0)	
- II	14 (20)	1 (4.8)	
- III	34 (48.6)	5 (23.8)	
- IV	9 (12.9)	15 (71.4)	
KRAS, *n* (%)	3 (4.3)	6 (28.6)	<0.01
MSI, *n* (%)	6 (8.6)	1 (4.8)	0.57
BRAF, *n* (%)	6 (8.4)	1 (4.8)	0.57

CB = clinical benefit, no-CB = no clinical benefit, *n* = number.

**Table 2:** Baseline metabolic parameters of the segmented primary tumors (*n* = 91 patients) stratified by clinical benefit (CB, *n* = 70) vs. no clinical benefit (No-CB, *n* = 21).

	CB (*n* = 70)	No-CB (*n* = 21)	*p*-Value
SUVmax			
- Median (range)	16.0 (10.9–20.8)	13.4 (12.2–17.5)	0.39
SUVmean			
- Median (range)	9.2 (6.5–11.8)	8.0 (6.7–10.3)	0.21
MTV (in cm^3^)			
- Median (range)	10.7 (6.4–19.3)	20.9 (15.7–36.4)	0.01
TLG			
- Median (range)	91.1 (47.1–182.9)	167.9 (100.5–310)	0.1

SUV = Standardized uptake value, MTV = metabolic tumor volume, TLG = total lesion glycolysis.

**Table 3:** Baseline morphological parameters of the segmented primary tumors (*n* = 91 patients) stratified by clinical benefit (CB, *n* = 70) vs. no clinical benefit (No-CB, *n* = 21).

	CB (*n* = 70)	Non-CB (*n* = 21)	*p*-Value
Morphology of the primary tumor, *n* (%)			
- Circular	38 (54.3)	19 (90.5)	
- Semi-circular	32 (45.7)	2 (9.5)	<0.01
Craniocaudal extension (in cm)			
- Median (range)	4.4 (2.9–5.5)	6.1 (4.7–7.8)	0.01
Volume of the primary tumor (in cm^3^)			
- Median (range)	10.8 (6.4–19.3)	21.1 (15.8–36.3)	0.01

**Table 4:** Logistic regression for clinical benefit.

Variable	HR	2.5%	97.5%
UICC stage, I–III vs. IV	0.06	0.02	0.23
Craniocaudal extension	0.74	0.57	0.96
Age	1.02	0.97	1.08
Gender	1.97	0.52	7.43

**Table 5:** Survival outcomes and mortality rates.

	CB (*n* = 70)	No-CB (*n* = 21)	*p*-Value
OS in months (SD)	36.1 (6.7)	29.1 (13.5)	0.03
PFS in months (SD)	36.1 (6.7)	14.4 (10.3)	<0.01
Observation time in months (SD)	36.6 (6.9)	37.6 (7.5)	0.58
Mortality rate	0.0%	48.0%	<0.01

SD: standard deviation.

**Table 6:** Univariate Cox regression for overall survival (OS).

Variable	HR	*p*-Value
Sex	0.29	0.06
UICC stage, I–III vs. IV	2.76	0.11
Craniocaudal extension	1.20	0.10
MTV PT	1.02	0.05

*n* = 91 patients included; number of deaths: 10.

**Table 7:** Univariate Cox regression for progression-free survival (PFS).

Variable	HR	*p*-Value
Sex	0.71	0.45
UICC stage, I–III vs. IV	9.37	<0.01
Craniocaudal extension	1.26	<0.01
MTV PT	1.02	<0.01

*n* = 91 patients included; number of progressions: 21.

**Table 8:** Multivariate Cox regression analysis for OS and PFS.

Model	Covariates	Exp (Coef)	95% CI (Log-Coef)	*p*-Value
OS	Sex	0.17	[−3.17; −0.32]	0.02
(AIC = 81.81; Conc. ind. = 0.77)	MTV PT	1.03	[0.01; 0.06]	<0.01
PFS	UICC	9.65	[1.30; 3.24]	<0.01
(AIC = 153.83; Conc. ind. = 0.82)	MTV PT	1.03	[0.01; 0.05]	<0.01

AIC = Akaike Information Criterion; Conc. ind. = concordance index.

## Data Availability

Data supporting the findings of this study are available from the corresponding author upon reasonable request.

## Abbreviations

AIC	Akaike Information Criterion
BMI	Body Mass Index
CB	Clinical Benefit
CI	Confidence Interval
CRC	Colorectal Cancer
CT	Computed Tomography
EKNZ	Ethics Committee for Northwest and Central Switzerland
ESMO	European Society for Medical Oncology
FDG	Fluorodeoxyglucose
GCP	Good Clinical Practice
HR	Hazard Ratio
LL	Log-Likelihood
LL-null	Null Log-Likelihood
MSI	Microsatellite Instability
MTV	Metabolic Tumor Volume
no-CB	No Clinical Benefit
OR	Odds Ratio
OS	Overall Survival
PET	Positron Emission Tomography
PFS	Progression-Free Survival
RECIST	Response Evaluation Criteria in Solid Tumors
ROC	Receiver Operating Characteristic
SUV	Standardized Uptake Value
SUVmax	Maximum Standardized Uptake Value
SUVmean	Mean Standardized Uptake Value
TLG	Total Lesion Glycolysis
UICC	Union for International Cancer Control
